# Crystal structure of (acetato-κ*O*)(ethanol-κ*O*)[(9*S*,17*S*,21*S*,29*S*)-9,17,21,29-tetra­hydroxy-18,30-dioxa­octa­cyclo­[18.10.0.0^2,7^.0^8,19^.0^9,17^.0^11,16^.0^21,29^.0^23,28^]triaconta-1,3,5,7,11(16),12,14,19,23(28),24,26-undeca­ene-10,22-dione-κ^3^ *O* ^18^,*O* ^21^,*O* ^22^]caesium ethanol monosolvate

**DOI:** 10.1107/S2056989016008860

**Published:** 2016-06-03

**Authors:** Ravell Bengiat, Maayan Gil, Asne Klein, Benny Bogoslavsky, Shmuel Cohen, Guy Yardeni, Israel Zilbermann, Joseph Almog

**Affiliations:** a Institute of Chemistry, The Hebrew University of Jerusalem, Jerusalem, 9190401, Israel; b Department of Chemistry, Nuclear Research Centre Negev, Beer Sheva, 84190, Israel

**Keywords:** crystal structure, vasarenes, ion-pair recognition, ninhydrin, supra­molecular complex

## Abstract

The title compound, [Cs(CH_3_COO)(C_28_H_16_O_8_)(C_2_H_5_OH)]·C_2_H_5_OH, is the product of the complexation between one vasarene analogue [**1**], bis ninhydrin naphthalene-1,3-diol and CsF, where the F^−^ ion has reacted with residual acetic acid (AcOH), to form a [**1**]·CsOAc complex. The inter­molecular inter­actions with the multiple oxygen-containing functional groups of the ligand, as well as O—H⋯O hydrogen bonds involving the ethanol solvent mol­ecules, stabilize the complex, forming a chain along [100]. Additional parallel-displaced π–π stacking, with an inter­planar distance of 3.669 (1) Å, connect several unit cells in a three-dimensional supra­molecular structure, though, the larger size of AcO^−^ (1.60 Å) compared to F^−^ (1.33 Å) prevents the tight packing that was once achieved with other vasarene complexes of CsF.

## Abstract 2

The title compound, C_28_H_16_O_8_·Cs^+^CH_3_O^−^·2CH_3_CH_2_OH, was formed in the supra­molecular reaction between a vasarene analogue and caesium fluoride, where the F^−^ ion has been replaced by acetate.

## Chemical context   

The supra­molecular reactions of ligands from the vasarene family with ion-pairs of type *M* ^+^F^−^, provided *M* is a large monovalent cation, have been studied extensively by our group in the past years (Almog *et al.*, 2009 ▸, 2012 ▸; Bengiat *et al.*, 2016**a* ▸,b* ▸,*c* ▸). The prerequisite regarding the size of the cation rests in the key role of the fluoride ion in initiating the complex formation (Bengiat *et al.*, 2016*b* ▸), though the contribution of the F^−^ ion to the stability of the complex once formed has yet to be explored. In several cases, however, the F^−^ ions have been absent from the final complex which contained acetate ions instead. This observation can be explained by the presence of acetic acid (AcOH) residues from the synthesis of the ligand, but the exact mechanism is still unknown. Here, we review the structure of the title complex and the effect of the AcO^−^ anion on its supra­molecular features.

## Structural commentary   

**Figure 1:**
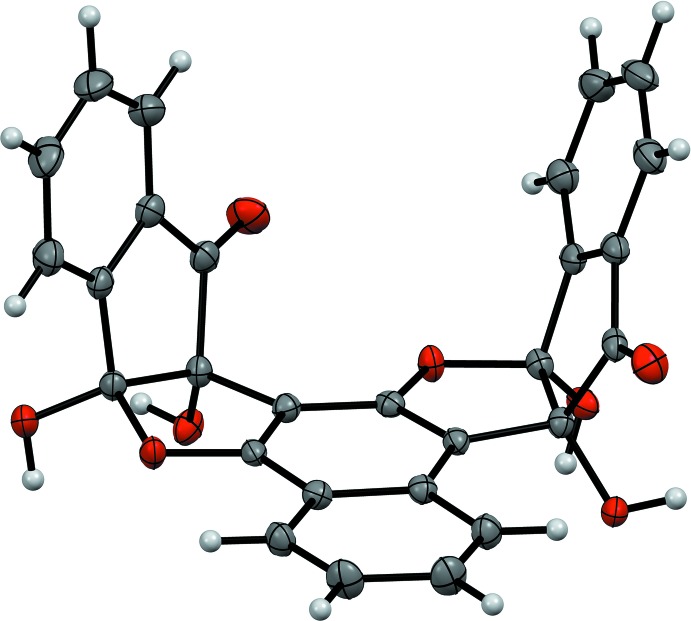
The mol­ecular structure of the bis ninhydrin naphthalene-1,3-diol ligand [**1**], showing 50% probability ellipsoids for non-H atoms. Solvent mol­ecules and the Cs^I^ ion have been omitted for clarity.

**Figure 2:**
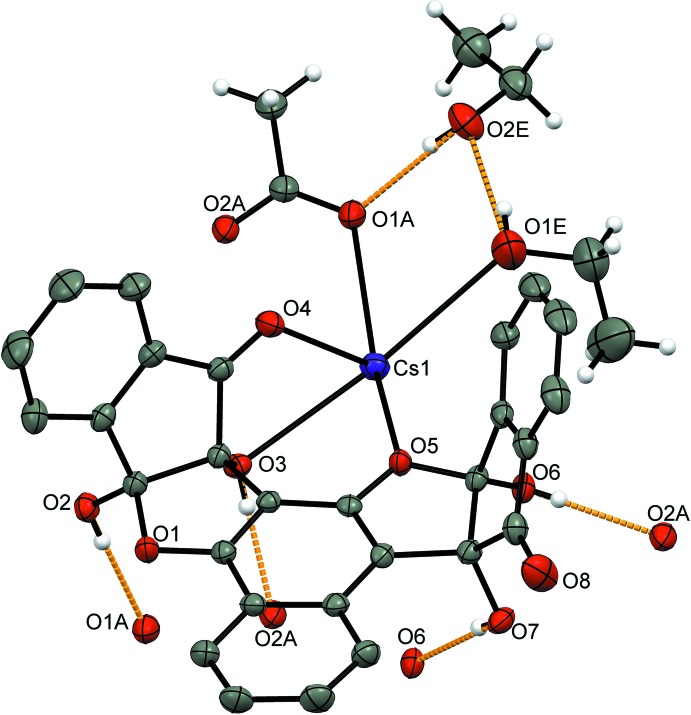
The mol­ecular structure of the bis ninhydrin naphthalene-1,3-diol [**1**] complex with CsOAc showing 50% probability ellipsoids for non-H atoms. Hydrogen bonding is represented by the orange dashed lines. Aromatic H atoms have been omitted for clarity. The codes for symmetry-related atoms are given in Table 1 ▸.

The complex was formed in the reaction of the bis ninhydrin naphthalene-1,3-diol ligand [**1**] (Fig. 1 ▸) with CsF. As mentioned earlier, we suggest that the presence of residual AcOH results in a selective precipitation with AcO^−^ rather than F^−^ in the final complex. Similar to the original vasarene complexes with CsF (Almog *et al.*, 2012 ▸; Bengiat *et al.*, 2016*b* ▸,*c* ▸), the Cs^+^ ion is stabilized by several inter­actions with the oxygen-containing functional groups of the ligand: hydroxyl (O3), carbonyl (O4) and etheric (O5), as well as by the additional EtOH solvate mol­ecule (O1*E*) and the acetate counter-ion (O1*A*) (Scheme, Fig. 2 ▸). 
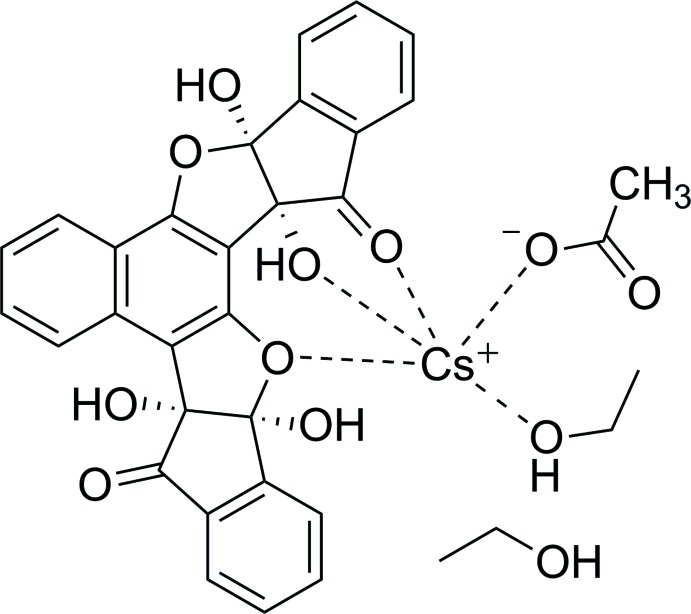


**Figure 3:**
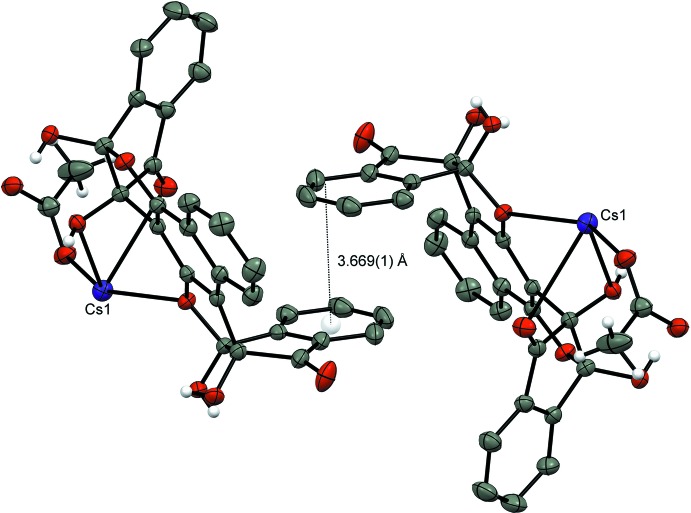
A fragment of the crystal packing of the [**1**]·CsOAc complex showing the parallel-displaced π–π stacking with an inter­planar distance of 3.669 Å at 50% probability ellipsoids for non-H atoms. Aromatic H atoms and solvent mol­ecules have been omitted for clarity.

**Figure 4:**
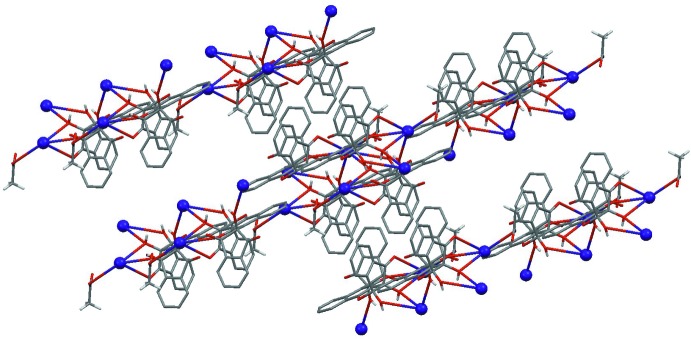
The crystal packing of the [**1**]·CsOAc complex showing 2 × 2 × 2 unit cells. Aromatic H atoms and solvent mol­ecules have been omitted for clarity.

Fig. 2 ▸ shows the hydrogen bonding between the different unit cells (Table 1 ▸) involving a second solvent mol­ecule of EtOH, O2*E*⋯H–O1*E* and O2*E*–H⋯O2*A*. Further stabilization of the lattice is achieved by the parallel-displaced π–π stacking between the aromatic rings of the ‘side-walls’ of ligands in different unit cells with an inter-planar distance of 3.669 (1) Å (Janiak, 2000 ▸) (Fig. 3 ▸). In other complexes of the vasarane analogues with CsF, there has been an alternating arrangement of ligand and salt layers, forming ‘salt channels’ that are held by supra­molecular inter­actions of hydrogen bonds, cation–π and metal coordination with the ligands (Bengiat *et al.*, 2016*b* ▸,*c* ▸). In this case, however, it is suggested that the difference in the ionic radius between the F^−^ (1.33 Å) and AcO^−^ (1.60 Å) (Shannon, 1976 ▸; Manku, 1980 ▸) results in steric hindrance that prevents the tight packing of the lattice (Figs. 4 ▸ and 5 ▸).

## Database survey   

The bowl-shaped compound formed upon reaction between ninhydrin and 1,3,5-benzene­triol was first reported by Kim and his co-workers (Na *et al.*, 2005 ▸), while other groups attempted similar reactions involving ninhydrin and poly­hydroxy aromatics (Kundu *et al.*, 2004 ▸; Mahmood *et al.*, 2011 ▸). Since then, the reaction has been thoroughly explored by our group, expanding the family of these ligands, which we have named vasarenes (Almog *et al.*, 2009 ▸; Gil *et al.*, 2014 ▸; Bengiat *et al.*, 2016*c* ▸,*d* ▸). A comprehensive study of the supra­molecular reactions of the vasarenes and their analogues with *M* ^+^F^−^ salts has also been carried out (Almog *et al.*, 2012 ▸; Bengiat *et al.*, 2016*a* ▸,*b* ▸,*c* ▸). However, this is the first time that a complex with an anion other than fluoride has been reported.

## Synthesis and crystallization   

The ligand [**1**] was synthesized according to a recently reported procedure (Bengiat *et al.* 2016*c* ▸) in a one-pot reaction in AcOH. Ligand [**1**] (151.0 mg, 0.314 mmol) was dissolved in warm EtOH (10 mL). An equivalent amount of CsF (50.1 mg, 0.329 mmol) was dissolved in warm EtOH (2 mL) with few drops of H_2_O_dist._ and added to the solution of [**1**]. Upon addition of the CsF solution an immediate color change to intense yellow was observed, later changing to bright orange. The mixture was left to crystallize at RT for a few days, forming a colorless crystalline precipitate suitable for single crystal X-ray diffraction.

## Refinement   

Crystal data, data collection and structure refinement details are summarized in Table 2 ▸. Hydroxyl H atoms of the ligand mol­ecules and H atoms of the EtOH mol­ecule were located in a different Fourier map and all H-atom parameters refined. Other H atoms were placed in calculated positions with C—H = 0.95 (aromatic), 0.99 (methyl­ene) and 0.98 Å (meth­yl), and refined in riding mode with *U*_iso_(H) = 1.2*U*_eq_(C) for aromatic and aliphatic H atoms and 1.5*U*_eq_(C) for the methyl H atoms.

## Supplementary Material

Crystal structure: contains datablock(s) I. DOI: 10.1107/S2056989016008860/lh5813sup1.cif

Structure factors: contains datablock(s) I. DOI: 10.1107/S2056989016008860/lh5813Isup2.hkl

CCDC reference: 1479382

Additional supporting information: crystallographic information; 3D view; checkCIF report

## Figures and Tables

**Figure 5:**
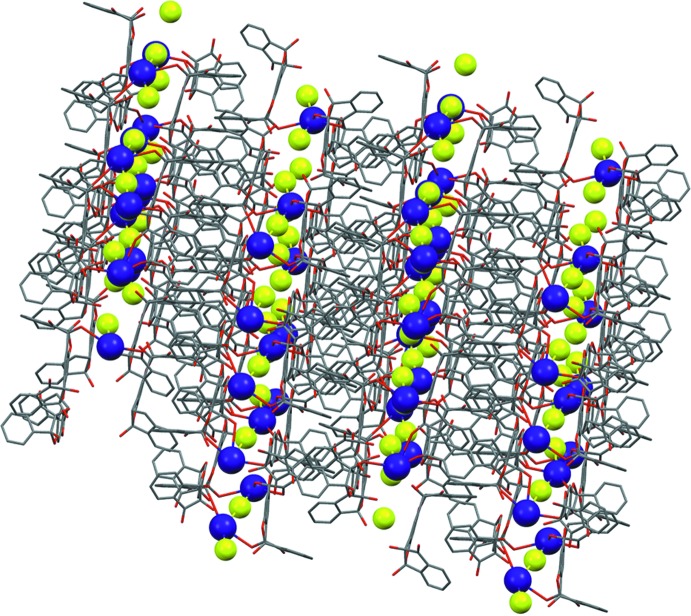
The crystal packing of the complex of bis ninhydrin 1,3-benzene­diol with CsF (Bengiat *et al.*, 2016*b* ▸) showing 2 × 2 × 2 unit cells. Aromatic H atoms and solvent mol­ecules have been omitted for clarity.

**Table 1:** Hydrogen-bond geometry (Å, °)

*D*—H⋯*A*	*D*—H	H⋯*A*	*D*⋯*A*	*D*—H⋯*A*
O2—H2*O*⋯O1*A* ^i^	0.72 (4)	1.98 (4)	2.704 (3)	175 (4)
O3—H3*O*⋯O2*A* ^i^	0.72 (3)	1.92 (3)	2.643 (3)	179 (4)
O6—H6*O*⋯O2*A* ^ii^	0.68 (4)	1.98 (4)	2.650 (3)	175 (4)
O7—H7*O*⋯O6^iii^	0.70 (4)	2.10 (4)	2.798 (3)	173 (4)
O1*E*—H1*E*⋯O2*E*	0.84 (4)	1.92 (5)	2.747 (4)	166 (4)
O2*E*—H2*E*⋯O1*A*	0.82 (4)	1.93 (4)	2.736 (3)	170 (4)

Symmetry codes: (i) 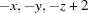; (ii) 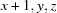; (iii) 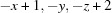.

**Table 2:** Experimental details

Crystal data	
Chemical formula	[Cs(C_2_H_3_O_2_)(C_28_H_16_O_8_)(C_2_H_6_O)]·C_2_H_6_O
*M*_r_	764.50
Crystal system, space group	Triclinic, *P* 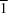
Temperature (K)	173
*a*, *b*, *c* (Å)	10.609 (2), 11.669 (2), 14.319 (2)
α, β, γ (°)	74.741 (2), 70.932 (2), 89.095 (2)
*V* (Å^3^)	1611.5 (4)
*Z*	2
Radiation type	Mo *K*α
μ (mm^−1^)	1.21
Crystal size (mm)	0.64 × 0.24 × 0.13

Data collection	
Diffractometer	Bruker SMART CCD
Absorption correction	Multi-scan (*SADABS*; Bruker, 2002 ▸)
*T*_min_, *T*_max_	0.511, 0.858
No. of measured, independent and observed [*I* > 2σ(*I*)] reflections	17584, 6917, 6818
*R*_int_	0.029
(sin θ/λ)_max_ (Å^−1^)	0.639

Refinement	
*R*[*F* ^2^ > 2σ(*F* ^2^)], *wR*(*F* ^2^), *S*	0.034, 0.085, 1.19
No. of reflections	6917
No. of parameters	451
H-atom treatment	H atoms treated by a mixture of independent and constrained refinement
Δρ_max_, Δρ_min_ (e Å^−3^)	1.72, −0.66

Computer programs: *SMART* and *SAINT* (Bruker, 2002 ▸), *SHELXS97*, *SHELXL97* and *SHELXTL* (Sheldrick, 2008 ▸) and *Mercury* (Macrae *et al.*, 2008 ▸).

## supplementary crystallographic information

### Crystal data

**Table:**

[Cs(C_2_H_3_O_2_)(C_28_H_16_O_8_)(C_2_H_6_O)]·C_2_H_6_O	*Z* = 2
*M**_r_* = 764.50	*F*(000) = 772
Triclinic, *P*1	*D*_x_ = 1.576 Mg m^−^^3^
Hall symbol: -P 1	Mo *K*α radiation, λ = 0.71073 Å
*a* = 10.609 (2) Å	Cell parameters from 7362 reflections
*b* = 11.669 (2) Å	θ = 2.1–28.1°
*c* = 14.319 (2) Å	µ = 1.21 mm^−^^1^
α = 74.741 (2)°	*T* = 173 K
β = 70.932 (2)°	Prisme, colorless
γ = 89.095 (2)°	0.64 × 0.24 × 0.13 mm
*V* = 1611.5 (4) Å^3^	

### Data collection

**Table:**

Bruker SMART CCD diffractometer	6917 independent reflections
Radiation source: fine-focus sealed tube	6818 reflections with *I* > 2σ(*I*)
Graphite monochromator	*R*_int_ = 0.029
φ and ω scans	θ_max_ = 27.0°, θ_min_ = 2.1°
Absorption correction: multi-scan (SADABS; Bruker, 2002)	*h* = −13→13
*T*_min_ = 0.511, *T*_max_ = 0.858	*k* = −14→14
17584 measured reflections	*l* = −18→18

### Refinement

**Table:**

Refinement on *F*^2^	Primary atom site location: structure-invariant direct methods
Least-squares matrix: full	Secondary atom site location: difference Fourier map
*R*[*F*^2^ > 2σ(*F*^2^)] = 0.034	Hydrogen site location: inferred from neighbouring sites
*wR*(*F*^2^) = 0.085	H atoms treated by a mixture of independent and constrained refinement
*S* = 1.19	*w* = 1/[σ^2^(*F*_o_^2^) + (0.0375*P*)^2^ + 1.213*P*] where *P* = (*F*_o_^2^ + 2*F*_c_^2^)/3
6917 reflections	(Δ/σ)_max_ = 0.007
451 parameters	Δρ_max_ = 1.72 e Å^−^^3^
0 restraints	Δρ_min_ = −0.66 e Å^−^^3^

### Special details

**Table:**

Geometry. All esds (except the esd in the dihedral angle between two l.s. planes) are estimated using the full covariance matrix. The cell esds are taken into account individually in the estimation of esds in distances, angles and torsion angles; correlations between esds in cell parameters are only used when they are defined by crystal symmetry. An approximate (isotropic) treatment of cell esds is used for estimating esds involving l.s. planes.
Refinement. Refinement of F^2^ against ALL reflections. The weighted R-factor wR and goodness of fit S are based on F^2^, conventional R-factors R are based on F, with F set to zero for negative F^2^. The threshold expression of F^2^ > 2sigma(F^2^) is used only for calculating R-factors(gt) etc. and is not relevant to the choice of reflections for refinement. R-factors based on F^2^ are statistically about twice as large as those based on F, and R- factors based on ALL data will be even larger.

### Fractional atomic coordinates and isotropic or equivalent isotropic displacement parameters (Å^2^)

**Table:**

	*x*	*y*	*z*	*U*_iso_*/*U*_eq_	
C1	0.2002 (2)	0.13669 (19)	1.17244 (17)	0.0175 (4)	
C2	0.1777 (2)	0.1095 (2)	1.27587 (18)	0.0199 (4)	
C3	0.2805 (2)	0.1067 (2)	1.31994 (18)	0.0209 (5)	
C4	0.4154 (2)	0.1316 (2)	1.25043 (19)	0.0213 (5)	
C5	0.4384 (2)	0.1527 (2)	1.14390 (18)	0.0194 (4)	
C6	0.3334 (2)	0.15554 (19)	1.10705 (17)	0.0181 (4)	
C7	0.2532 (3)	0.0832 (2)	1.4266 (2)	0.0264 (5)	
H7	0.1638	0.0640	1.4725	0.032*	
C8	0.3557 (3)	0.0882 (3)	1.4641 (2)	0.0316 (6)	
H8	0.3375	0.0731	1.5359	0.038*	
C9	0.4886 (3)	0.1158 (3)	1.3959 (2)	0.0327 (6)	
H9	0.5586	0.1213	1.4226	0.039*	
C10	0.5189 (3)	0.1348 (2)	1.2928 (2)	0.0273 (5)	
H10	0.6095	0.1502	1.2489	0.033*	
C11	−0.0331 (2)	0.1068 (2)	1.26594 (18)	0.0204 (4)	
C12	0.0699 (2)	0.1299 (2)	1.15298 (18)	0.0186 (4)	
C13	0.0413 (2)	0.2535 (2)	1.09573 (19)	0.0211 (5)	
C14	−0.0449 (2)	0.3084 (2)	1.17370 (19)	0.0230 (5)	
C15	−0.0903 (2)	0.2250 (2)	1.26866 (19)	0.0225 (5)	
C16	−0.1744 (3)	0.2560 (3)	1.3541 (2)	0.0307 (6)	
H16	−0.2059	0.1995	1.4195	0.037*	
C17	−0.2108 (3)	0.3720 (3)	1.3405 (2)	0.0404 (7)	
H17	−0.2678	0.3952	1.3979	0.048*	
C18	−0.1660 (3)	0.4555 (3)	1.2449 (3)	0.0404 (7)	
H18	−0.1931	0.5344	1.2380	0.049*	
C19	−0.0827 (3)	0.4247 (2)	1.1601 (2)	0.0320 (6)	
H19	−0.0521	0.4811	1.0946	0.038*	
C21	0.5131 (2)	0.1982 (2)	0.96238 (18)	0.0196 (4)	
C22	0.5672 (2)	0.1622 (2)	1.05530 (18)	0.0208 (5)	
C23	0.6674 (2)	0.2676 (2)	1.0343 (2)	0.0259 (5)	
C24	0.6405 (2)	0.3677 (2)	0.9577 (2)	0.0251 (5)	
C25	0.5503 (2)	0.3302 (2)	0.91796 (19)	0.0220 (5)	
C26	0.5078 (3)	0.4087 (2)	0.8450 (2)	0.0290 (5)	
H26	0.4445	0.3833	0.8192	0.035*	
C27	0.5618 (3)	0.5262 (3)	0.8112 (2)	0.0364 (6)	
H27	0.5346	0.5824	0.7614	0.044*	
C28	0.6550 (3)	0.5628 (2)	0.8490 (2)	0.0380 (7)	
H28	0.6919	0.6431	0.8232	0.046*	
C29	0.6945 (3)	0.4858 (2)	0.9226 (2)	0.0340 (6)	
H29	0.7568	0.5119	0.9490	0.041*	
Cs1	0.191153 (14)	0.124796 (13)	0.862730 (11)	0.02585 (7)	
O1	0.04685 (16)	0.08256 (15)	1.33507 (13)	0.0226 (3)	
O2	−0.12922 (17)	0.01494 (17)	1.29727 (14)	0.0243 (4)	
H2O	−0.099 (3)	−0.041 (3)	1.299 (3)	0.034 (10)*	
O3	0.06070 (17)	0.04548 (16)	1.10108 (13)	0.0212 (3)	
H3O	0.093 (3)	−0.005 (3)	1.122 (2)	0.021 (8)*	
O4	0.08513 (19)	0.29564 (16)	1.00382 (14)	0.0289 (4)	
O5	0.36872 (15)	0.17430 (15)	1.00338 (12)	0.0197 (3)	
O6	0.55495 (19)	0.13249 (16)	0.89154 (14)	0.0229 (4)	
H6O	0.623 (4)	0.137 (3)	0.874 (3)	0.031 (10)*	
O7	0.63353 (19)	0.05673 (17)	1.06295 (15)	0.0262 (4)	
H7O	0.584 (3)	0.013 (3)	1.071 (3)	0.031 (10)*	
O8	0.7534 (2)	0.2654 (2)	1.07278 (17)	0.0417 (5)	
C1A	−0.0995 (3)	0.2127 (2)	0.7471 (2)	0.0292 (5)	
C2A	−0.1497 (4)	0.3298 (3)	0.7045 (3)	0.0605 (11)	
H2A1	−0.1226	0.3911	0.7310	0.091*	
H2A2	−0.2475	0.3214	0.7254	0.091*	
H2A3	−0.1116	0.3529	0.6293	0.091*	
O1A	0.02108 (18)	0.19599 (17)	0.70590 (15)	0.0322 (4)	
O2A	−0.18031 (18)	0.13858 (16)	0.82327 (15)	0.0302 (4)	
C1E	0.4943 (4)	0.3196 (4)	0.5980 (3)	0.0606 (10)	
H1E1	0.5492	0.3891	0.5951	0.073*	
H1E2	0.4918	0.3275	0.5281	0.073*	
C2E	0.5549 (5)	0.2099 (5)	0.6319 (3)	0.0800 (15)	
H2E1	0.5524	0.1999	0.7027	0.120*	
H2E2	0.6479	0.2146	0.5869	0.120*	
H2E3	0.5049	0.1418	0.6290	0.120*	
O1E	0.3619 (3)	0.3185 (3)	0.6666 (2)	0.0576 (7)	
H1E	0.319 (4)	0.340 (4)	0.625 (3)	0.063 (13)*	
C3E	0.2150 (3)	0.3851 (3)	0.4560 (2)	0.0419 (7)	
H3E1	0.2817	0.3271	0.4387	0.050*	
H3E2	0.2551	0.4653	0.4122	0.050*	
C4E	0.0925 (4)	0.3560 (3)	0.4324 (3)	0.0483 (8)	
H4E1	0.0530	0.2762	0.4748	0.072*	
H4E2	0.1180	0.3584	0.3596	0.072*	
H4E3	0.0271	0.4146	0.4474	0.072*	
O2E	0.1861 (2)	0.3825 (2)	0.56062 (18)	0.0436 (5)	
H2E	0.131 (4)	0.329 (4)	0.600 (3)	0.056 (12)*	

### Atomic displacement parameters (Å^2^)

**Table:**

	*U*^11^	*U*^22^	*U*^33^	*U*^12^	*U*^13^	*U*^23^
C1	0.0179 (10)	0.0144 (10)	0.0202 (11)	0.0014 (8)	−0.0073 (8)	−0.0036 (8)
C2	0.0186 (10)	0.0173 (10)	0.0221 (11)	0.0024 (8)	−0.0057 (9)	−0.0038 (9)
C3	0.0225 (11)	0.0184 (11)	0.0210 (11)	0.0028 (8)	−0.0088 (9)	−0.0025 (9)
C4	0.0210 (11)	0.0171 (11)	0.0248 (12)	0.0021 (8)	−0.0088 (9)	−0.0026 (9)
C5	0.0169 (10)	0.0157 (10)	0.0244 (12)	−0.0002 (8)	−0.0060 (9)	−0.0048 (9)
C6	0.0203 (11)	0.0130 (10)	0.0201 (11)	0.0014 (8)	−0.0064 (9)	−0.0034 (8)
C7	0.0258 (12)	0.0272 (12)	0.0239 (12)	0.0045 (10)	−0.0082 (10)	−0.0035 (10)
C8	0.0355 (14)	0.0364 (15)	0.0242 (13)	0.0058 (11)	−0.0151 (11)	−0.0041 (11)
C9	0.0318 (14)	0.0391 (15)	0.0314 (14)	0.0029 (11)	−0.0194 (11)	−0.0053 (12)
C10	0.0229 (12)	0.0284 (13)	0.0300 (13)	0.0023 (10)	−0.0118 (10)	−0.0034 (10)
C11	0.0173 (10)	0.0225 (11)	0.0206 (11)	0.0017 (8)	−0.0057 (9)	−0.0055 (9)
C12	0.0162 (10)	0.0185 (11)	0.0206 (11)	0.0015 (8)	−0.0052 (8)	−0.0058 (9)
C13	0.0185 (10)	0.0198 (11)	0.0256 (12)	0.0024 (8)	−0.0091 (9)	−0.0053 (9)
C14	0.0210 (11)	0.0230 (12)	0.0267 (12)	0.0053 (9)	−0.0091 (9)	−0.0083 (10)
C15	0.0178 (10)	0.0238 (12)	0.0278 (12)	0.0019 (9)	−0.0079 (9)	−0.0099 (10)
C16	0.0255 (12)	0.0344 (14)	0.0301 (14)	0.0027 (10)	−0.0030 (10)	−0.0131 (11)
C17	0.0386 (16)	0.0392 (16)	0.0418 (17)	0.0116 (13)	−0.0039 (13)	−0.0211 (14)
C18	0.0453 (17)	0.0292 (15)	0.0471 (18)	0.0155 (12)	−0.0113 (14)	−0.0167 (13)
C19	0.0349 (14)	0.0252 (13)	0.0349 (15)	0.0079 (11)	−0.0108 (11)	−0.0080 (11)
C21	0.0154 (10)	0.0189 (11)	0.0234 (11)	0.0004 (8)	−0.0038 (8)	−0.0074 (9)
C22	0.0179 (10)	0.0184 (11)	0.0247 (12)	0.0014 (8)	−0.0065 (9)	−0.0043 (9)
C23	0.0201 (11)	0.0275 (13)	0.0276 (13)	−0.0035 (9)	−0.0042 (9)	−0.0078 (10)
C24	0.0224 (11)	0.0237 (12)	0.0256 (12)	−0.0026 (9)	−0.0023 (9)	−0.0079 (10)
C25	0.0193 (11)	0.0197 (11)	0.0227 (12)	0.0022 (8)	−0.0006 (9)	−0.0069 (9)
C26	0.0291 (13)	0.0259 (13)	0.0275 (13)	0.0048 (10)	−0.0055 (10)	−0.0048 (10)
C27	0.0418 (16)	0.0249 (13)	0.0324 (15)	0.0093 (11)	−0.0030 (12)	−0.0036 (11)
C28	0.0459 (17)	0.0169 (12)	0.0388 (16)	−0.0022 (11)	0.0014 (13)	−0.0066 (11)
C29	0.0333 (14)	0.0278 (14)	0.0358 (15)	−0.0069 (11)	−0.0012 (11)	−0.0128 (12)
Cs1	0.02629 (10)	0.02675 (10)	0.02485 (10)	0.00161 (6)	−0.01049 (6)	−0.00509 (6)
O1	0.0181 (8)	0.0271 (9)	0.0194 (8)	0.0011 (6)	−0.0046 (6)	−0.0032 (7)
O2	0.0197 (8)	0.0216 (9)	0.0273 (9)	−0.0019 (7)	−0.0034 (7)	−0.0053 (7)
O3	0.0224 (8)	0.0179 (8)	0.0249 (9)	0.0026 (7)	−0.0096 (7)	−0.0066 (7)
O4	0.0327 (9)	0.0260 (9)	0.0232 (9)	0.0066 (7)	−0.0065 (7)	−0.0026 (7)
O5	0.0154 (7)	0.0229 (8)	0.0191 (8)	0.0000 (6)	−0.0040 (6)	−0.0050 (6)
O6	0.0181 (9)	0.0226 (9)	0.0262 (9)	0.0009 (7)	−0.0022 (7)	−0.0099 (7)
O7	0.0199 (9)	0.0219 (9)	0.0353 (10)	0.0055 (7)	−0.0085 (7)	−0.0064 (8)
O8	0.0324 (10)	0.0480 (13)	0.0437 (12)	−0.0132 (9)	−0.0196 (9)	−0.0014 (10)
C1A	0.0294 (13)	0.0248 (13)	0.0281 (13)	0.0048 (10)	−0.0062 (10)	−0.0029 (10)
C2A	0.0462 (19)	0.0423 (19)	0.056 (2)	0.0219 (15)	0.0086 (16)	0.0148 (16)
O1A	0.0236 (9)	0.0261 (9)	0.0361 (10)	0.0034 (7)	−0.0023 (8)	−0.0003 (8)
O2A	0.0249 (9)	0.0228 (9)	0.0331 (10)	0.0031 (7)	−0.0015 (7)	−0.0019 (8)
C1E	0.050 (2)	0.068 (3)	0.048 (2)	0.0046 (18)	−0.0024 (16)	−0.0071 (19)
C2E	0.092 (3)	0.105 (4)	0.049 (2)	0.049 (3)	−0.025 (2)	−0.033 (2)
O1E	0.0424 (13)	0.0750 (19)	0.0410 (14)	0.0083 (13)	−0.0057 (11)	−0.0026 (13)
C3E	0.0445 (17)	0.0315 (15)	0.0380 (17)	0.0020 (12)	−0.0029 (13)	−0.0036 (13)
C4E	0.055 (2)	0.0434 (18)	0.0427 (18)	0.0000 (15)	−0.0115 (15)	−0.0115 (15)
O2E	0.0448 (13)	0.0393 (12)	0.0366 (12)	−0.0098 (10)	−0.0067 (10)	−0.0015 (10)

### Geometric parameters (Å, º)

**Table:**

C1—C2	1.369 (3)	C26—C27	1.392 (4)
C1—C6	1.399 (3)	C26—H26	0.9500
C1—C12	1.504 (3)	C27—C28	1.392 (5)
C2—O1	1.360 (3)	C27—H27	0.9500
C2—C3	1.423 (3)	C28—C29	1.368 (5)
C3—C7	1.409 (3)	C28—H28	0.9500
C3—C4	1.434 (3)	C29—H29	0.9500
C4—C5	1.417 (3)	Cs1—O7^i^	3.0101 (18)
C4—C10	1.422 (3)	Cs1—O3	3.1163 (18)
C5—C6	1.377 (3)	Cs1—O1E	3.121 (3)
C5—C22	1.512 (3)	Cs1—O4	3.1441 (18)
C6—O5	1.364 (3)	Cs1—O3^ii^	3.1744 (17)
C7—C8	1.369 (4)	Cs1—O1A	3.257 (2)
C7—H7	0.9500	Cs1—O5	3.3263 (16)
C8—C9	1.412 (4)	Cs1—O2^ii^	3.3650 (19)
C8—H8	0.9500	Cs1—Cs1^ii^	4.9310 (5)
C9—C10	1.361 (4)	Cs1—H3O	3.43 (3)
C9—H9	0.9500	O2—Cs1^ii^	3.3650 (19)
C10—H10	0.9500	O2—H2O	0.72 (4)
C11—O2	1.368 (3)	O3—Cs1^ii^	3.1744 (17)
C11—O1	1.474 (3)	O3—H3O	0.72 (3)
C11—C15	1.503 (3)	O6—H6O	0.68 (4)
C11—C12	1.584 (3)	O7—Cs1^i^	3.0101 (18)
C12—O3	1.402 (3)	O7—H7O	0.70 (4)
C12—C13	1.539 (3)	C1A—O1A	1.258 (3)
C13—O4	1.207 (3)	C1A—O2A	1.261 (3)
C13—C14	1.478 (3)	C1A—C2A	1.507 (4)
C14—C15	1.385 (4)	C2A—H2A1	0.9800
C14—C19	1.392 (4)	C2A—H2A2	0.9800
C15—C16	1.390 (3)	C2A—H2A3	0.9800
C16—C17	1.383 (4)	C1E—O1E	1.424 (4)
C16—H16	0.9500	C1E—C2E	1.462 (6)
C17—C18	1.391 (5)	C1E—H1E1	0.9900
C17—H17	0.9500	C1E—H1E2	0.9900
C18—C19	1.379 (4)	C2E—H2E1	0.9800
C18—H18	0.9500	C2E—H2E2	0.9800
C19—H19	0.9500	C2E—H2E3	0.9800
C21—O6	1.385 (3)	O1E—H1E	0.84 (4)
C21—O5	1.452 (3)	C3E—O2E	1.420 (4)
C21—C25	1.508 (3)	C3E—C4E	1.509 (5)
C21—C22	1.572 (3)	C3E—H3E1	0.9900
C22—O7	1.405 (3)	C3E—H3E2	0.9900
C22—C23	1.540 (3)	C4E—H4E1	0.9800
C23—O8	1.207 (3)	C4E—H4E2	0.9800
C23—C24	1.472 (4)	C4E—H4E3	0.9800
C24—C25	1.389 (4)	O2E—H1E	1.92 (5)
C24—C29	1.398 (4)	O2E—H2E	0.82 (4)
C25—C26	1.387 (4)		
			
C2—C1—C6	117.4 (2)	O3—Cs1—O4	54.35 (5)
C2—C1—C12	110.02 (19)	O1E—Cs1—O4	97.22 (7)
C6—C1—C12	132.4 (2)	O7^i^—Cs1—O3^ii^	99.87 (5)
O1—C2—C1	114.2 (2)	O3—Cs1—O3^ii^	76.77 (5)
O1—C2—C3	121.7 (2)	O1E—Cs1—O3^ii^	130.26 (7)
C1—C2—C3	124.1 (2)	O4—Cs1—O3^ii^	105.68 (5)
C7—C3—C2	122.5 (2)	O7^i^—Cs1—O1A	143.97 (5)
C7—C3—C4	120.5 (2)	O3—Cs1—O1A	123.70 (5)
C2—C3—C4	117.0 (2)	O1E—Cs1—O1A	71.34 (6)
C5—C4—C10	123.7 (2)	O4—Cs1—O1A	101.41 (5)
C5—C4—C3	118.6 (2)	O3^ii^—Cs1—O1A	61.26 (5)
C10—C4—C3	117.7 (2)	O7^i^—Cs1—O5	58.07 (5)
C6—C5—C4	120.9 (2)	O3—Cs1—O5	61.73 (4)
C6—C5—C22	108.2 (2)	O1E—Cs1—O5	93.43 (6)
C4—C5—C22	130.6 (2)	O4—Cs1—O5	61.33 (4)
O5—C6—C5	115.3 (2)	O3^ii^—Cs1—O5	136.30 (4)
O5—C6—C1	122.8 (2)	O1A—Cs1—O5	156.10 (4)
C5—C6—C1	121.9 (2)	O7^i^—Cs1—O2^ii^	95.85 (5)
C8—C7—C3	119.9 (2)	O3—Cs1—O2^ii^	124.63 (4)
C8—C7—H7	120.1	O1E—Cs1—O2^ii^	86.84 (7)
C3—C7—H7	120.1	O4—Cs1—O2^ii^	146.21 (5)
C7—C8—C9	120.0 (2)	O3^ii^—Cs1—O2^ii^	50.73 (4)
C7—C8—H8	120.0	O1A—Cs1—O2^ii^	48.17 (5)
C9—C8—H8	120.0	O5—Cs1—O2^ii^	152.22 (4)
C10—C9—C8	121.6 (2)	O7^i^—Cs1—Cs1^ii^	86.79 (4)
C10—C9—H9	119.2	O3—Cs1—Cs1^ii^	38.81 (3)
C8—C9—H9	119.2	O1E—Cs1—Cs1^ii^	162.27 (5)
C9—C10—C4	120.3 (2)	O4—Cs1—Cs1^ii^	78.79 (4)
C9—C10—H10	119.9	O3^ii^—Cs1—Cs1^ii^	37.97 (3)
C4—C10—H10	119.9	O1A—Cs1—Cs1^ii^	92.36 (3)
O2—C11—O1	108.65 (18)	O5—Cs1—Cs1^ii^	99.56 (3)
O2—C11—C15	113.02 (19)	O2^ii^—Cs1—Cs1^ii^	87.23 (3)
O1—C11—C15	107.72 (19)	O7^i^—Cs1—H3O	63.4 (5)
O2—C11—C12	116.42 (19)	O3—Cs1—H3O	11.5 (5)
O1—C11—C12	106.32 (17)	O1E—Cs1—H3O	150.9 (5)
C15—C11—C12	104.20 (19)	O4—Cs1—H3O	63.9 (5)
O3—C12—C1	114.48 (18)	O3^ii^—Cs1—H3O	78.1 (5)
O3—C12—C13	110.29 (19)	O1A—Cs1—H3O	131.6 (5)
C1—C12—C13	110.50 (18)	O5—Cs1—H3O	58.5 (5)
O3—C12—C11	115.78 (18)	O2^ii^—Cs1—H3O	121.5 (5)
C1—C12—C11	100.72 (18)	Cs1^ii^—Cs1—H3O	41.2 (5)
C13—C12—C11	104.30 (18)	C2—O1—C11	107.60 (17)
O4—C13—C14	127.9 (2)	C11—O2—Cs1^ii^	123.60 (14)
O4—C13—C12	124.6 (2)	C11—O2—H2O	110 (3)
C14—C13—C12	107.4 (2)	Cs1^ii^—O2—H2O	68 (3)
C15—C14—C19	121.5 (2)	C12—O3—Cs1	116.40 (13)
C15—C14—C13	110.2 (2)	C12—O3—Cs1^ii^	128.36 (13)
C19—C14—C13	128.3 (2)	Cs1—O3—Cs1^ii^	103.23 (5)
C14—C15—C16	120.6 (2)	C12—O3—H3O	105 (2)
C14—C15—C11	112.3 (2)	Cs1—O3—H3O	110 (2)
C16—C15—C11	127.1 (2)	Cs1^ii^—O3—H3O	90 (2)
C17—C16—C15	117.7 (3)	C13—O4—Cs1	118.88 (15)
C17—C16—H16	121.1	C6—O5—C21	106.32 (17)
C15—C16—H16	121.1	C6—O5—Cs1	130.11 (13)
C16—C17—C18	121.7 (3)	C21—O5—Cs1	122.45 (12)
C16—C17—H17	119.2	C21—O6—H6O	107 (3)
C18—C17—H17	119.2	C22—O7—Cs1^i^	156.51 (16)
C19—C18—C17	120.6 (3)	C22—O7—H7O	104 (3)
C19—C18—H18	119.7	Cs1^i^—O7—H7O	92 (3)
C17—C18—H18	119.7	O1A—C1A—O2A	124.0 (2)
C18—C19—C14	117.9 (3)	O1A—C1A—C2A	118.1 (2)
C18—C19—H19	121.1	O2A—C1A—C2A	118.0 (2)
C14—C19—H19	121.1	C1A—C2A—H2A1	109.5
O6—C21—O5	104.28 (18)	C1A—C2A—H2A2	109.5
O6—C21—C25	114.35 (19)	H2A1—C2A—H2A2	109.5
O5—C21—C25	110.50 (18)	C1A—C2A—H2A3	109.5
O6—C21—C22	115.48 (19)	H2A1—C2A—H2A3	109.5
O5—C21—C22	107.60 (18)	H2A2—C2A—H2A3	109.5
C25—C21—C22	104.53 (18)	C1A—O1A—Cs1	114.97 (17)
O7—C22—C5	113.06 (19)	O1E—C1E—C2E	110.5 (4)
O7—C22—C23	108.95 (19)	O1E—C1E—H1E1	109.6
C5—C22—C23	115.6 (2)	C2E—C1E—H1E1	109.6
O7—C22—C21	115.02 (19)	O1E—C1E—H1E2	109.6
C5—C22—C21	100.30 (17)	C2E—C1E—H1E2	109.6
C23—C22—C21	103.53 (19)	H1E1—C1E—H1E2	108.1
O8—C23—C24	127.4 (2)	C1E—C2E—H2E1	109.5
O8—C23—C22	125.3 (2)	C1E—C2E—H2E2	109.5
C24—C23—C22	107.3 (2)	H2E1—C2E—H2E2	109.5
C25—C24—C29	120.6 (3)	C1E—C2E—H2E3	109.5
C25—C24—C23	110.6 (2)	H2E1—C2E—H2E3	109.5
C29—C24—C23	128.8 (2)	H2E2—C2E—H2E3	109.5
C26—C25—C24	121.5 (2)	C1E—O1E—Cs1	131.0 (2)
C26—C25—C21	127.6 (2)	C1E—O1E—H1E	101 (3)
C24—C25—C21	110.9 (2)	Cs1—O1E—H1E	111 (3)
C25—C26—C27	117.3 (3)	O2E—C3E—C4E	112.8 (3)
C25—C26—H26	121.4	O2E—C3E—H3E1	109.0
C27—C26—H26	121.4	C4E—C3E—H3E1	109.0
C26—C27—C28	121.1 (3)	O2E—C3E—H3E2	109.0
C26—C27—H27	119.5	C4E—C3E—H3E2	109.0
C28—C27—H27	119.5	H3E1—C3E—H3E2	107.8
C29—C28—C27	121.5 (3)	C3E—C4E—H4E1	109.5
C29—C28—H28	119.2	C3E—C4E—H4E2	109.5
C27—C28—H28	119.2	H4E1—C4E—H4E2	109.5
C28—C29—C24	118.0 (3)	C3E—C4E—H4E3	109.5
C28—C29—H29	121.0	H4E1—C4E—H4E3	109.5
C24—C29—H29	121.0	H4E2—C4E—H4E3	109.5
O7^i^—Cs1—O3	74.74 (5)	C3E—O2E—H1E	120.1 (14)
O7^i^—Cs1—O1E	110.43 (6)	C3E—O2E—H2E	113 (3)
O3—Cs1—O1E	148.09 (7)	H1E—O2E—H2E	96 (3)
O7^i^—Cs1—O4	113.67 (5)		

Symmetry codes: (i) −*x*+1, −*y*, −*z*+2; (ii) −*x*, −*y*, −*z*+2.

### Hydrogen-bond geometry (Å, º)

**Table:**

*D*—H···*A*	*D*—H	H···*A*	*D*···*A*	*D*—H···*A*
O2—H2*O*···O1*A*^ii^	0.72 (4)	1.98 (4)	2.704 (3)	175 (4)
O3—H3*O*···O2*A*^ii^	0.72 (3)	1.92 (3)	2.643 (3)	179 (4)
O6—H6*O*···O2*A*^iii^	0.68 (4)	1.98 (4)	2.650 (3)	175 (4)
O7—H7*O*···O6^i^	0.70 (4)	2.10 (4)	2.798 (3)	173 (4)
O1*E*—H1*E*···O2*E*	0.84 (4)	1.92 (5)	2.747 (4)	166 (4)
O2*E*—H2*E*···O1*A*	0.82 (4)	1.93 (4)	2.736 (3)	170 (4)

Symmetry codes: (i) −*x*+1, −*y*, −*z*+2; (ii) −*x*, −*y*, −*z*+2; (iii) *x*+1, *y*, *z*.
